# Supplementary material for: Imaging strategies for follow-up during adjuvant nivolumab in esophageal cancer: A multicenter retrospective cohort study
Source: PLoS One. 2026 Jun 2;21(6):e0350105. doi: 10.1371/journal.pone.0350105 (PMC13229294; doi:10.1371/journal.pone.0350105)
Supplement: Attachment — Submitted filename: Point-to-point rebuttal_05042026.docx [file pone.0350105.s003.docx]

**Point-to-point response letter**

**_________________________________________ Reviewer 1 _______________________________________**

Reviewer #1: Dear Authors,
Thank you for submitting this manuscript evaluating CT imaging strategies during adjuvant nivolumab treatment for esophageal cancer. This study addresses a clinically relevant question that has practical implications for patient follow-up after curative-intent treatment. The multi-center design and real-world data complement the pivotal CheckMate 577 trial findings. I have carefully reviewed your manuscript against PLOS ONE publication criteria. Below I provide detailed feedback organized by major issues, minor issues, and specific recommendations for improvement.

*We would like to thank the Reviewer for the thorough and constructive comments. We have made every effort to address the recommendations and believe that these revisions have improved the manuscript. For clarity and readability, we have combined some points where appropriate. Please find below our responses to the Reviewer’s comments and a summary of the changes made to the manuscript.*

**MAJOR ISSUES**
**MAJOR ISSUE 1: DATA AVAILABILITY STATEMENT CONTRADICTION**
There is a contradiction between your submission form responses and the manuscript text regarding data availability: Submission Form (Page 4-5) and information files." Manuscript Text (Lines 283-286):
"Due to the nature of the study, in which exception consent was obtained from participants, the data used in this research cannot be made publicly available. Data sharing is not permitted as per the consent agreement."

*Thank you for your feedback regarding the data availability statement. We have updated the statement in the manuscript (page 12) as requested. However, we are unable to change the selection in the submission portal, as we do not have access to this option in the revision phase. After contact with the Editor of PLOS ONE, we changed the statement as follows:*

*"Data cannot be shared publicly due to ethical restrictions related to participant consent. The ethics approval (MEC2023-0631) specified that individual-level data would not be publicly released. Requests for de‑identified, aggregated data (including summary statistics and additional subgroup analyses upon request) may be directed to the Department of Internal Oncology at Erasmus University Medical Center via* [*interne.oncologie@erasmusmc.nl*](mailto:interne.oncologie@erasmusmc.nl) *and will require approval from the Medical Research Ethical Committee of Erasmus University Medical Center Rotterdam."*

**MAJOR ISSUE 2: CONCLUSIONS EXCEED WHAT THE DATA CAN SUPPORT**
The manuscript title, abstract, and conclusions make claims that exceed what a retrospective, non-randomized observational study can establish: Title: "Optimizing imaging strategies..." Abstract (Lines 46-47): "Routine CT imaging every 4 months, starting at 4 months after surgery, effectively detects recurrences during adjuvant nivolumab treatment while reducing unnecessary imaging." Conclusion (Lines 268-274): Implies the 4-month interval is the recommended or optimal strategy. However, your data show that both imaging strategies produced equivalent outcomes: Q3M group (n=67):
- 12 patients (17.9%) developed recurrence
- 8 of 12 recurrences (66.7%) detected by routine CT
- Diagnostic yield: 5% at 3 months, 8% at 6 months, 3% at 9 months
Q4M group (n=84):
- 15 patients (17.9%) developed recurrence
- 10 of 15 recurrences (66.7%) detected by routine CT
- Diagnostic yield: 9% at 4 months, 8% at 8 months
Both strategies showed nearly identical recurrence rates (~18%) and detection rates (~67%). Your study design cannot determine which is superior because:
1. Patients were not randomized to imaging intervals
2. Institutional practice determined interval (potential confounding)
3. Sample sizes preclude detecting meaningful differences
4. No formal statistical comparison between groups was performed

RECOMMENDATION:
1. Revise the title to:
"Characterizing imaging strategies for adjuvant nivolumab in esophageal cancer: a multi-center retrospective cohort study" OR "Imaging follow-up patterns during adjuvant nivolumab for esophageal
cancer: real-world data from three Dutch centers"
2. Revise the abstract conclusion (Lines 46-51) to:
"In this retrospective cohort, routine CT imaging at 4-month intervals detected the majority of on-treatment recurrences. The gradual decline in disease-free survival suggests that recurrences are distributed over
time rather than clustered at specific time points. These real-world data may help inform, but cannot definitively establish, optimal follow-up intervals. Prospective studies comparing imaging strategies
are needed."
3. Revise the Discussion conclusion (Lines 268-279) to explicitly state: "This observational study cannot determine the optimal imaging interval. Our findings describe current practice patterns and outcomes, which may inform clinical decision-making pending prospective comparative studies."
4. Add a statement acknowledging that both Q3M and Q4M showed equivalent outcomes in your cohort (12/67 vs 15/84 recurrences; 8/12 vs 10/15 detected by routine CT), and that selection of imaging interval should consider institutional resources, patient preferences, and clinical judgment rather than presumed superiority of one approach.

*Thank you for your thoughtful and detailed feedback. We fully agree that, given the retrospective and non-randomized design of our study, we cannot definitively establish the superiority of any imaging regimen. We appreciate your careful consideration of the study’s limitations, including the lack of randomization, potential confounding by institutional practice, and limited sample sizes, which preclude formal statistical comparison between groups.*

*In response, we have revised the manuscript to ensure that our claims are closely aligned with the data and presented in a more descriptive manner. Specifically:*

- *The title and abstract have been updated to reflect the observational nature of the study and to avoid overstating the conclusions as following:*

*“Imaging strategies for follow-up during adjuvant nivolumab in esophageal cancer: a multicenter retrospective cohort study”*

- *The manuscript now explicitly states that both imaging strategies (Q3M and Q4M) produced nearly identical recurrence and detection rates, and that selection of imaging interval should be guided by institutional resources, patient preferences, and clinical judgment rather than presumed superiority. We revised the conclusion in the abstract as follows:*

*“Routine baseline CT imaging did not detect recurrences, while routine imaging during adjuvant nivolumab identified the majority of recurrences. The gradual decline in disease-free survival suggests that recurrences are evenly distributed over time, supporting a routine imaging interval, such as every 3 or 4 months as used in our study. These real-world data may help inform clinicians, and future studies can further evaluate optimal imaging intervals.”*

- *The discussion and conclusion sections have been revised to clarify that our findings describe real-world practice patterns and outcomes, which may inform clinical decision-making, but cannot determine the optimal imaging interval. We also acknowledge the need for prospective comparative studies to address this question.*

*“In conclusion, this retrospective cohort study showed that 18% of patients with esophageal cancer, previously treated with neoadjuvant chemoradiotherapy and resection, developed recurrent disease during adjuvant treatment with nivolumab. This observational study cannot determine the optimal imaging interval. Our findings describe current practice patterns and outcomes, which may inform clinical decision-making pending prospective comparative studies.*

*Routine baseline CT scans appear to have limited utility, as early recurrences were predominantly detected based on clinical symptoms. However, considering the timing of recurrences, follow-up intervals of 3–4 months for early recurrences and 7–8 months for late on-treatment recurrences could be reasonable options. Reducing the number of scans requires careful consideration of recurrence detection, imaging frequency, feasibility, and healthcare costs. At the same time, follow-up imaging remains essential, as many recurrences detected by routine CT scans were asymptomatic. Given the high costs of nivolumab and the potential for severe adverse effects, timely detection of recurrence is crucial to avoid unnecessary exposure to treatment in patients who may not benefit. The choice of routine imaging intervals should be guided by institutional resources, patient preferences, and clinical judgment.”*

**MAJOR ISSUE 3: MULTIPLE DATA DISCREPANCIES**
Several numerical inconsistencies were identified that must be corrected:
DISCREPANCY 1 - Patient Age: Line 160: "The nivolumab cohort included 151 patients, with a median age of 69 years (IQR, 60-72)..." Table 1: "Median age (range) - year: 66 [60-72]". The IQR values match (60-72), but the median differs by 3 years (69 vs 66).
DISCREPANCY 2 - Lymph Node Status P-Value: Line 193-194: "both the early and late recurrence groups had a higher proportion of patients with ≥N1 disease post-surgery (p = 0.039)" Table 4: Shows p = 0.021 for "Pathological lymph-node status post-surgery" These p-values differ substantially (0.039 vs 0.021) for what appears to be the same analysis. I suspect Table 1 (66 years) is correct for age based on the IQR, and Table 4 (p=0.021) may be correct for the lymph node analysis, with the text containing typographical errors - but please verify from original data.

*We apologize for the inconsistencies in the representation of the data. We have carefully reviewed the original dataset and corrected the manuscript as follows:*

- ***Patient Age:*** *The correct median age is 66 years (IQR 60–72), as shown in Table 1. The text has been updated to reflect this value.*
- ***Lymph Node Status P-Value:*** *The correct p-value for pathological lymph-node status post-surgery is 0.021, as indicated in Table 4. The text has been revised accordingly.*

*We have ensured that all numerical values in the manuscript are consistent with the original data. Thank you for bringing these discrepancies to our attention.*

**MAJOR ISSUE 4: STROBE CHECKLIST NOT PROVIDED**
Lines 100-101 state: "Data collection and reporting followed the Strengthening the Reporting of Observational Studies in Epidemiology (STROBE) guidelines."

*We apologize that the STROBE checklist was not readily found within the submission. It was originally uploaded as Supporting Information, which may have caused it to be overlooked among the supplemental files. To ensure clarity, we have re-uploaded the checklist as supplemental data and revised the manuscript as follows:*

*"Data collection and reporting followed the Strengthening the Reporting of Observational Studies in Epidemiology (STROBE) guidelines. (15) A completed STROBE checklist is provided as Supporting Information (S1 Checklist)."*

**MAJOR ISSUE 5: INCOMPLETE LIMITATIONS DISCUSSION**
While the Discussion acknowledges some limitations (Lines 228-231, 238-243), several important limitations are not explicitly addressed:
1. INABILITY TO COMPARE IMAGING STRATEGIES: The non-randomized design means institutional preference determined imaging interval, not random assignment. Any differences (or lack thereof) could reflect selection bias or confounding rather than true equivalence.
2. LEAD-TIME BIAS: Earlier or more frequent detection of recurrence through imaging may not translate to improved outcomes. Patients detected earlier appear to survive longer from diagnosis simply because diagnosis occurred earlier, not because of any true survival benefit.
3. DETECTION BIAS: The 67% detection rate by routine CT may be influenced by the timing of scans. Some "symptom-driven" detections may have been caught on the next routine scan regardless.
4. LIMITED GENERALIZABILITY: The cohort is predominantly male (82%, 124/151), adenocarcinoma (86%, 130/151), from Dutch academic centers. Results may not generalize to other populations, histologies, or healthcare settings.
5. EVOLVING TREATMENT LANDSCAPE: With FLOT becoming standard for adenocarcinoma, the population eligible for nCRT + adjuvant nivolumab is changing, potentially limiting future applicability.

*Thank you for these valuable suggestions. We have specifically addressed the issues of selection bias, lead-time bias, detection bias, limited generalizability, and the evolving treatment landscape, as suggested by the reviewer. We have extended the limitations section in the manuscript to address the points raised, as follows (page 10):*

*“Several limitations must be considered when interpreting the findings of this study. First, detection bias may have occurred, as some recurrences detected during scheduled imaging were already clinically suspected, potentially overestimating the detection rate in both strategies. Second, the non-randomized design and variability in institutional practices prevented a formal comparison between the Q3M and Q4M imaging strategies, as any differences between groups may reflect confounding factors rather than inherent differences. The generalizability of the results may be limited due to the homogeneity of the cohort, which was predominantly male (82%) and mainly comprised of adenocarcinoma patients (86%) from Dutch centers. These factors may limit the applicability of the findings to other populations, histologies, or healthcare settings. Lastly, while early detection may help spare patients from unnecessary treatments and their associated risks, timely detection of recurrence through routine imaging does not necessarily translate into better patient outcomes. Based on our data, we cannot determine whether detecting recurrence 1-2 months earlier with one strategy (Q3M vs Q4M) would lead to improved outcomes, such as greater opportunities for salvage surgery or other curative interventions, especially considering that the majority of recurrences were distant.”*

**MINOR ISSUES**
**MINOR ISSUE 2: PD-L1 ANALYSIS - CONFUSING PRESENTATION AND INTERPRETATION**
The PD-L1 subgroup analysis in Table 4 has confusing presentation:
Table 4 shows for "PD-L1 CPS <5": The more clinically meaningful comparison is among patients WITH available
PD-L1 data: - Early recurrence: 7/11 (63.6%) had CPS <5 - No recurrence: 8/29 (27.6%) had CPS <5
Additionally, this is based on very small numbers (only 41 patients had PD-L1 data available per Line 195), making the p=0.001 finding potentially unreliable.
RECOMMENDATION:
1. Clarify the Table 4 presentation - specify whether percentages refer to:
- Proportion of those with available PD-L1 data, OR Proportion of the total group
2. Consider presenting both: "7/11 (63.6% of those tested; 33.3% of early recurrence group)"
3. More explicitly frame as hypothesis-generating in the text (Lines 195-196):
"In an exploratory analysis limited by small sample size (n=41 with available PD-L1 data), patients with early recurrence were more likely to have CPS <5 (7/11, 63.6%) compared to those without recurrence
(8/29, 27.6%), p=0.001. This finding should be interpreted with caution given the small numbers and requires validation in larger cohorts."

*Thank you for this comment. We added these detailed numbers in Table 4, and added the more*

*cautious interpretation to the Discussion as requested.*

**MINOR ISSUE 3: CT READING METHODOLOGY NOT SPECIFIED**

The manuscript does not describe how CT scans were interpreted: Single radiologist or consensus

reading? Were readers blinded to clinical information? Were standardized criteria used for defining recurrence

on imaging?

*Thank you for this comment. We agree that the methodology for CT scan interpretation should be clarified. As the CT scans were performed as part of routine clinical care, they were interpreted by board-certified radiologists according to standard clinical practice at each participating center. No standardized research criteria or consensus readings were applied, and readers were not blinded to clinical information. We have clarified this in the revised manuscript as follows (Method section, page 4-5):*

*"CT scans were performed as part of routine clinical care and were retrospectively reviewed for the purposes of this study. Board-certified radiologists interpreted the scans in accordance with standard clinical practice at each center, and blinding was not applied. Recurrence was defined as the presence of new lesions suspicious for malignancy. In cases where clinical symptoms were later confirmed by imaging, the date of the CT scan was considered the date of recurrence."*

**MINOR ISSUE 4: MISSING COMPARISON TABLE FOR SELECTION BIAS ASSESSMENT**
Of 229 eligible patients, only 151 (66%) received nivolumab. The 78 patients who did not receive treatment may differ systematically from treated patients. Understanding these differences is important for assessing selection bias.
The reasons for not starting treatment are listed (Lines 155-157):
- Poor performance status: n=22, patient preference: n=33, contraindications: n=5, post-operative death: n=7, pre-treatment recurrence: n=11. However, baseline characteristics of these patients are not compared to treated patients. RECOMMENDATION:
Add a supplementary table (S1 Table) comparing baseline characteristics
between: - Patients who received nivolumab (n=151); - Eligible patients who did not receive nivolumab (n=78)
Include: age, sex, histology, stage, pathological response, performance status, and reason for not receiving treatment. This allows readers to assess whether the treated cohort is representative of the broader eligible population.

*Thank you for this suggestion. We agree that understanding the selection process is important for interpreting our findings. However, the primary aim of our study was to describe recurrence patterns specifically in patients who received adjuvant nivolumab. Comparing baseline characteristics between treated and untreated patients would introduce substantial selection bias, bias by indication, and immortal time bias, as patients who did not receive nivolumab often had clear reasons for exclusion (e.g., poor performance status, early recurrence, or contraindications). These differences reflect clinical decision-making rather than random allocation, and such a comparison would not address the study’s primary objective, nor would it allow for meaningful conclusions regarding the effect of nivolumab.*

*Nevertheless, to provide transparency regarding the selection process, we have clarified the inclusion criteria and reasons for non-treatment in the Results section (page 5), and we have added a new flowchart (Figure 1) illustrating the selection of the study population. We hope this approach allows readers to understand the real-world eligibility and treatment patterns without introducing additional bias through inappropriate comparisons. We changed the manuscript as follows:*

*“A total of 451 patients underwent resection for esophageal or esophageal junction carcinoma between September 1, 2021, and October 31, 2023 (Figure 1). Of these, 98 patients (22%) were excluded based on treatment prior to surgery, including eighteen patients (5%) who participated in clinical trials with additional systemic therapy: tocilizumab in the BASALT trial (NCT04554771(17)), trastuzumab/pertuzumab in the TRAP-2 trial (NCT05188313(18)) and nivolumab in the SANO-3 study (NCT05491616(19)).*

*Of the remaining 353 patients, 78% received nCRT with carboplatin/paclitaxel. After surgery, 74 patients (21%) had a complete pathological response, and 20 (6%) were excluded due to microscopic irradical resection (R1). Thirty patients (8%) were excluded due to missing data. A total of 229 (65%) patients were assessed for eligibility for adjuvant nivolumab, of whom 151 (43%) received at least one cycle.*

*The main reasons for not starting adjuvant treatment were poor performance status (n=22), patient preference (n=33), and symptomatic recurrence (n=11) detected before starting adjuvant treatment.”*

**MINOR ISSUE 5: TIMING CONVENTION INCONSISTENCY**
Lines 117-118 define DFS from "start of adjuvant nivolumab," but some analyses and discussion reference time from surgery. This can cause confusion when interpreting timing of events.
For example:
- DFS is from nivolumab start (Line 117)
- But nivolumab starts ≤16 weeks after surgery (Line 117-118)
- Table 3 shows "Time (months)" without specifying reference point
- Discussion mentions "4 months after surgery" (Line 46)
RECOMMENDATION:
1. Clearly state the reference time point (surgery vs. nivolumab start) for each analysis
2. Consider adding a note that nivolumab was initiated within 16 weeks of surgery (per protocol), so timepoints approximately align
3. In Table 3 header, specify: "Time (months from nivolumab initiation)"
4. In abstract/conclusions, clarify whether "4 months after surgery" means 4 months from surgery or 4 months from nivolumab start

*Thank you for addressing this important point, we apologies for the inconsistency. We do agree that defining the index date is of great importance. We tried to clarify this as following:*

- *Page 5 (Method section): “DFS, defined as the time (in months) from the start of adjuvant nivolumab to disease recurrence or death, whichever occurred first. Adjuvant nivolumab was initiated within 16 weeks after esophagectomy for every patient, per protocol. Unless otherwise specified, all timepoints and analyses are referenced from the start of adjuvant nivolumab.”*
- *Page 17 (Table 3): “Time (months from nivolumab initiation)”*

**OTHER MINOR ISSUES**

MINOR ISSUE 1: TYPOGRAPHICAL AND FORMATTING ERRORS

MINOR ISSUE 6: CONFIDENCE INTERVALS FOR KEY ESTIMATES
Key percentages are reported without confidence intervals:
- On-treatment recurrence: 27/151 (18%) Detection by routine CT: 18/27 (67%); - 12-month DFS: 75%
This makes it difficult to assess precision of estimates, which is particularly important given the relatively small sample size. RECOMMENDATION: Add 95% confidence intervals for: on-treatment recurrence rate: 27/151 = 17.9% (95% CI: 12.1-24.9%); detection by routine CT: 18/27 = 66.7% (95% CI: 46.0-83.5%); DFS at 4, 8, and 12 months (from Kaplan-Meier analysis)

MINOR ISSUE 7: REFERENCE TO UNPUBLISHED DATA
Lines 68-72 reference "Updated results from CheckMate 577, presented at the 2025 ASCO Annual Meeting" with overall survival data. Reference 12 cites this as a conference abstract.
MINOR ISSUE 8: DIAGNOSTIC YIELD CALCULATION CLARIFICATION
The diagnostic yield calculations (Lines 180-185) could be clearer: Q3M group: - 5% (3/62) at 3 months; - 8% (4/51) at 6 months; - 3% (1/32) at 9 months; - 17% (3/18) at 12 months. Q4M group: - 9% (6/68) at 4 months; - 8% (4/49) at 8 months
The denominators (62, 51, 32, 18, 68, 49) represent patients who had CT scans at each timepoint, not the total cohort. This is appropriate but could be stated more explicitly.
RECOMMENDATION:
Add clarification: "Diagnostic yield was calculated as the number of recurrences detected divided by the number of patients who underwent routine CT scanning at each timepoint. Denominators decrease over time due to prior recurrence, treatment discontinuation, or study end."

*We thank the reviewer for the helpful minor comments. We have addressed all points as requested, and all minor comments have been incorporated into the revised manuscript as requested.*

- ***MINOR ISSUE 1: Typographical and formatting errors***

*We have carefully revised the manuscript and corrected all typographical and formatting errors as requested.*

- ***MINOR ISSUE 6: Confidence intervals for key estimates*** *Results section (page 7): We have added 95% confidence intervals for the on-treatment recurrence rate, the proportion of recurrences detected by routine CT, and for DFS at 4, 8, and 12 months (from Kaplan-Meier analysis) in the Results section.*
- ***MINOR ISSUE 7: Reference to unpublished data*** *Introduction section (page 3): “These data have been presented in abstract form only and have not yet undergone peer review. Final conclusions regarding overall survival benefit should await full publication”*
- ***MINOR ISSUE 8: Diagnostic yield calculation clarification*** *We have clarified in the Method and Result section (page 7) that diagnostic yield was calculated as the number of recurrences detected divided by the number of patients who underwent routine CT scanning at each timepoint. The denominators decrease over time due to prior recurrence, treatment discontinuation, or study end.*

**QUESTIONS REQUIRING CLARIFICATION**
1. PROTOCOL DEVIATIONS: Were there instances where patients deviated from their assigned imaging protocol (e.g., Q3M patient receiving scan at 4 months due to scheduling)? If so, how were these handled in the analysis?

*There were occasional deviations from the assigned imaging protocol, such as scans performed outside the scheduled interval (e.g., a Q3M patient receiving a scan at 4 months due to scheduling logistics). In most cases, the intended scan intervals were maintained. These minor deviations were not specifically corrected for in the analysis, unless a patient consistently followed a different scan protocol. We expect that these deviations did not significantly impact our outcomes.*

2. SYMPTOM-DRIVEN VS. ROUTINE SCANS: How were "symptom-driven" scans (Table 2, n=3, 11%) distinguished from routine scans in your data collection? Was this based on documented clinical indication, or timing relative to protocol?

*Symptom-driven scans were distinguished from routine scans based on the documented clinical indication in the CT request and review of the medical records. If the CT request stated a clinical suspicion or was performed outside the routine schedule, it was classified as symptom-driven. We clarified this in the Method section (page 5):*

*"CT scans were classified as symptom-driven if the clinical indication was documented in the scan request or medical record, and the scan was performed outside the routine protocol."*

3. PRE-TREATMENT RECURRENCES: For the 11 patients with symptomatic recurrence before starting nivolumab, what was the median time from surgery to recurrence detection? This information would help contextualize the potential value of baseline imaging.

*The median time between surgery and disease recurrence detection was 7.9 weeks (IQR, 4-10). All these patients were symptomatic, and indication for imaging was based on clinical suspicion of recurrent disease. For patients that underwent routine baseline CT scanning prior to nivolumab initiation (n= 69), median time between surgery and first CT scan was 10.6 weeks (IQR 7.9-13.0).*

*To address this point, we added the following sentences:*

- *Result section (page 8): “In 69 (46%) patients, a baseline CT scan was performed before the start of adjuvant nivolumab, with no recurrences detected. Median time between surgery and baseline CT was 10.6 weeks (IQR 7.9-13.0).”*
- *Discussion section (page 9):*

*“The median time from surgery to recurrence detection in these symptomatic patients was 7.9 weeks (IQR 4–10). For patients who underwent routine baseline CT scanning prior to nivolumab initiation (n = 69), the median time between surgery and baseline CT was 10.6 weeks (IQR 7.9–13.0), which largely overlapped with or was even later than the timing of symptomatic recurrences. This suggests that routine baseline CT would not have detected these recurrences earlier than clinical evaluation.”*

4. LOSS TO FOLLOW-UP: Were any patients lost to follow-up during the study period? If so, how many and how were they handled in survival analyses?

*Data on outpatient clinic registration was available for all patients during nivolumab treatment. Patients with missing data on nivolumab administration or imaging details were excluded from the analyses (see Flowchart in Figure 1). If patients were lost to follow-up after discontinuing nivolumab but before the end of the administrative follow-up period, they were censored at their last known clinic visit in the disease-free survival (DFS) analysis, as described in the statistical methods section (page 5).*

5. IMAGING PROTOCOLS: Were there differences in CT protocols between institutions (e.g., contrast, slice thickness, body regions imaged)? Could this affect detection sensitivity?

*It is possible that minor differences in CT protocols existed between institutions, such as variations in contrast use and slice thickness. However, all three participating hospitals routinely performed high-quality thorax and abdomen CT scans according to standard clinical practice. Given the consistency in imaging protocols and quality across centers, we do not expect that these differences significantly affected detection sensitivity or influenced our results*.

6. P-VALUE DISCREPANCY: Please clarify the correct p-value for the lymph node status analysis - is it 0.039 (Line 193-194) or 0.021 (Table 4)?
7. VERIFICATION: Given the multiple numerical discrepancies identified, can you confirm that all statistics have been independently verified against the source database?

*Thank you for your comment. We can confirm that all data have been independently verified against the source database. The data now align between the tables and the text, and we appreciate your careful review.*

**_________________________________________ Reviewer 2 _______________________________________**

Reviewer #2: To the authors:

Thank you for submitting your manuscript entitled “Optimizing imaging strategies for adjuvant nivolumab in esophageal cancer: the planning of scanning,” which evaluates real‑world imaging strategies during adjuvant nivolumab therapy for esophageal or gastroesophageal junction cancer following neoadjuvant chemoradiotherapy (nCRT) and R0 resection. By comparing 3‑monthly versus 4‑monthly CT‑based follow‑up schedules and their respective diagnostic yields for recurrence detection, the study suggests that a 4‑monthly scanning interval may balance the need for timely detection with the aims of minimizing radiation exposure and controlling healthcare costs. Although this study is of considerable interest, there are several issues I would like to raise prior to publication.

**1. Exclusions for nivolumab:** The study reports that 74 patients (21%) with a complete pathological response (ypT0N0) and 20 patients (6%) with microscopic irradical resection (R1) after surgery were excluded from the nivolumab cohort. Could you further clarify the specific follow‑up strategies, including imaging intervals, implemented for these patients who did not receive adjuvant nivolumab? In particular, were follow‑up intervals extended for ypT0N0 patients or shortened for R1 patients, and what were the actual surveillance strategies used in routine practice for these groups?

*We appreciate the reviewer’s suggestion to clarify the follow-up strategies for patients who did not receive adjuvant nivolumab. As noted, the 74 patients (21%) with a complete pathological response (ypT0N0) and the 20 patients (6%) with microscopic irradical resection (R1) after surgery were excluded from the nivolumab cohort based on the criteria outlined in the methods section. Therefore, detailed follow-up data for these patients were not available for analysis in our study. We acknowledge that surveillance strategies may differ for these groups in routine practice, with potentially extended intervals for ypT0N0 patients and shortened intervals for R1 patients, but this could not be assessed in our cohort, and is beyond the scope of this analysis.*

**2. Imaging strategy for high‑risk esophageal cancer patients:** The study identifies ≥ypN1 disease and PD‑L1 CPS <5 as factors associated with a higher risk of on‑treatment recurrence, although PD‑L1 data were available only in a subset of patients. Could you clarify whether the proposed strategy of “routine CT imaging every 4 months” has been specifically evaluated in these higher‑risk subgroups? Alternatively, should a more frequent interval, such as a Q3M strategy, be considered for such patients to ensure sufficiently timely detection of recurrence?

*Thank you for this highly clinically relevant question. It is well established in the literature that patients with post-surgical lymph node involvement (≥ypN1) have a higher risk of disease recurrence [source]. In our cohort, pathological lymph-node status post-surgery was significantly associated with recurrence (p = 0.021).* *However, the proposed strategy of routine CT imaging every 4 months (Q4M) was not specifically evaluated in these higher risk subgroups. Our study was not powered to formally compare imaging intervals within risk strata, and the observational design precludes definitive recommendations for more frequent imaging in high-risk patients. Additionally, a substantial proportion of recurrences in our cohort were detected based on clinical symptoms rather than routine imaging, independent of the scan schedule (33%). Therefore, it remains unclear whether a more frequent scan regimen would lead to earlier detection or improved outcomes in these patients. Nevertheless, heightened clinical awareness and vigilance in high-risk subgroups is essential, and prospective studies are needed to evaluate the optimal imaging strategy for these patients.*

*We added the following sentence to the discussion (page 9):*

*“Further research is needed to confirm whether low PD-L1 expression and nodal positivity can serve as reliable biomarkers of early on-treatment recurrence, and to determine whether more frequent imaging strategies could facilitate earlier detection and improve patient outcomes. Heightened clinical awareness and close monitoring remain particularly important for these high-risk patients.”*

**3. Comparison of Q4M vs Q3M interval strategy based on the diagnostic yield of CT scans:** The study reports that, in the Q3M group, the diagnostic yield for recurrence detection was 5% at 3 months, 8% at 6 months, 3% at 9 months, and 17% at 12 months. In the Q4M group, the diagnostic yield was 9% at 4 months and 8% at 8 months, and no routine 12‑month CT scans were performed. To definitively conclude that Q4M is “superior” to Q3M, the following questions need to be addressed:
3‑1. Rationale for the absence of 12‑month CT scans in the Q4M group: Please provide a clear explanation for why routine CT scans were not performed at 12 months for patients in the Q4M group. Understanding the rationale for this omission is important for a complete comparison of the longer‑term performance of the two imaging strategies.

*While timely detection at 12 months may influence treatment decisions moving forward, the variations in the timing of CT scans—both prior to treatment initiation and after completion of treatment—reflect the absence of formal guidelines recommending routine imaging intervals. In the Q4M regimen, after the last treatment, patients typically undergo follow-up evaluations according to clinical guidelines, which include clinical assessments and imaging only when symptoms arise. In clinical practice, once the final cycle of treatment has been completed, the goal of routine CT imaging—primarily to prevent unnecessary treatment toxicity and its associated costs—has limited value. Of course, it can be argued that timely detection at the end of treatment may influence treatment decisions thereafter. The variation in daily practice highlights the difficulty of balancing the costs of imaging and healthcare resources with the potential benefits of early detection.*

**3‑2. Statistical significance of differences in diagnostic yield:** Given the observed diagnostic yields in the Q3M and Q4M groups, was any statistical analysis performed to assess whether there is a significant difference in recurrence detection rates between these schedules at comparable time points? A formal statistical comparison would strengthen any claims regarding the relative performance of the two strategies.

*Thank you for your insightful comment regarding the statistical analysis of the diagnostic yield in the Q3M and Q4M groups. After carefully reviewing your comment and those of the other reviewers, we have decided not to conduct a formal statistical comparison of the two regimens.*

*The main reasons for this decision are the lack of shared timepoints between the two groups, the observational nature of the study, and the complexities introduced by symptomatic detection and subsequent scan timing. Given these factors, we believe that it would not be possible to draw meaningful or statistically valid comparisons between the two strategies.*

**3‑3. Impact of delayed detection on patient outcomes:** The Q4M schedule inherently introduces an approximately one‑month delay in the first routine evaluation (3 vs 4 months) and a two‑month delay in the second evaluation (6 vs 8 months) compared with the Q3M schedule. Could you discuss whether such delays might adversely affect patients’ chances for potentially curative or salvage therapy, or otherwise worsen prognosis? Timely identification of recurrence is important to initiate salvage treatment as soon as possible. Therefore, a thorough discussion of the clinical implications of these detection delays is warranted.

*We cannot determine whether detecting recurrence 1-2 months earlier with one strategy (Q3M vs Q4M) would lead to improved outcomes, such as greater opportunities for salvage surgery or other curative interventions, especially considering that the majority of recurrences was distant. It may be expected that locoregional recurrences, which are potentially curable, are detected more often based on clinical symptoms than routine imaging. However, we cannot definitively state this based on our data. This has been incorporated into the limitations section of the discussion.*

*We’ve addressed this point in the Discussion (page 10) as follows:*

*“Lastly, while early detection may help spare patients from unnecessary treatments and their associated risks, timely detection of recurrence through routine imaging does not necessarily translate into better patient outcomes. Based on our data, we cannot determine whether detecting recurrence 1-2 months earlier with one strategy (Q3M vs Q4M) would lead to improved outcomes, such as greater opportunities for salvage surgery or other curative interventions, especially considering that the majority of recurrences was distant.”*

**_________________________________________ Reviewer 3 _______________________________________**

Reviewer #3: Conceptual issue:

The paper considers two protocols, Q3M which has CAT scans at 3, 6, 9 and 12 months, and Q4M which has CAT scans at 4 and 8 months. The authors would like to reach some conclusion about whether Q4M is competitive with Q3M for detection of relapse.

Unfortunately I think this is close to impossible due to the limitations of the data. There is no shared timepoint between the two protocols, so at any time where they could be compared, one has been scanned more recently than the other. Relapses which are not caught by scan are sometimes caught because they become
symptomatic, so that later scans are not done at all, introducing further complication.

*We would like to thank the Reviewer for their thoughtful comments regarding the limitations of comparing the Q3M and Q4M protocols. We fully agree that, due to the lack of shared timepoints, the observational nature of the study, and the complexities introduced by symptomatic detection and subsequent scan timing, it is not possible to formally compare clinical outcomes between the two strategies.*

*After careful consideration and in response to feedback from multiple reviewers, we have decided to remove all direct statistic comparisons between Q3M and Q4M from the manuscript. The revised version now presents the findings in a purely descriptive manner, without implying superiority or equivalence of either protocol in terms of clinical outcomes. Accordingly, we have updated the title and tempered the conclusions throughout the manuscript (abstract, discussion; also see Major Point 2 of Reviewer 1 on page 2 and 3 of this response letter).*

The authors divide detected reccurrences into early (0-6 months) and late (7-12 months). However, since Q4M cannot detect recurrences in months 5 or 6 until month 8, an identical recurrence will be "early" in Q3M and "late" in Q4M. And the "late" category does not include months 9-12 for Q4M at all as those can never
be caught by scanning, since no 12 month scan is done. Perhaps there is a sophisticated type of statistical analysis which could work here. This reviewer is not an expert in trial statistics. However, no such analysis has been attempted.

*Thank you for this insightful comment regarding the categorization of early and late recurrences. We agree that the division into early (0–6 months) and late (7–12 months) recurrences is influenced by differences in scan timing between the Q3M and Q4M protocols. However, it is important to note that while the majority of recurrences (67%) was detected during routine imaging, a substantial proportion (33%) were identified based on clinical suspicion, which could occur at any time, independent of scheduled scans.*

*To address this point, we have clarified in the revised manuscript that both routine and symptom-driven recurrences were included in our analyses. We also explain our rationale for using a 6-month cutoff for early recurrences, as this timeframe is clinically relevant for timely detection and intervention. Furthermore, when identifying predictors for early recurrence, we did not stratify by scan protocol, but instead analyzed the entire cohort to maximize statistical power and clinical applicability.*

The only hope I see of getting any answer to the relative effectiveness of Q3M vs Q4M is Monte Carlo simulation where various assumptions can be made about the underlying distribution of events, they can be subjected to Q3M and Q4M, and the resulting simulated data can be compared to the real data. The results of the study may be worth presenting anyway--there is a good deal of information here. But I am very uncomfortable with almost all conclusions drawn by the authors. Certainly the statement that Q4M seems adequate is not based in the data.

*We appreciate the reviewer’s suggestion regarding the use of Monte Carlo simulation to assess the relative effectiveness of Q3M vs Q4M imaging strategies. While such an approach could potentially provide insights under certain conditions, it would require strong assumptions about the underlying distribution of recurrence events and patient characteristics, which are difficult to justify given the non-randomized, observational nature of our study and the influence of real-world clinical practices. Additionally, the presence of confounding factors and variability in patient management further complicate the modeling of these strategies.*

*Given these limitations, we have chosen to focus on descriptive analysis and to avoid drawing conclusions about the relative effectiveness or adequacy of Q3M versus Q4M. In response to Reviewer feedback, we have revised the manuscript to ensure that our claims are closely aligned with the data, and we have tempered statements regarding the adequacy of the Q4M protocol. We now emphasize that our findings describe real-world practice patterns and recurrence detection rates, and that prospective, randomized studies or simulation-based approaches would be needed to more definitively compare imaging strategies.*

Presentational issues:
(1) There are discrepancies both within the front matter (likely generated by the submission website) and between front matter and paper. These need to be checked and corrected. Specifically:
- Front matter: states no funding; paper: lists funding sources
- Front matter: answers "yes" to "are data available?"; instructions specifically state that "by request" should be treated as a "no" answer and explained. Paper: states that data are only available by request
- Both paper and front matter state that there are no known competing financial interests, and then go on to list, in detail, potential competing interests. I don't understand this: it appears contradictory.

Thank you for your insightful comments regarding the discrepancies between the front matter and the paper. We agree that there were inconsistencies that could lead to confusion. Below are the updates made to the revised manuscript:

- The front matter initially stated that there was no funding, while the paper listed funding sources. To resolve this, we have updated the front matter to reflect the funding sources accurately, aligning it with the paper

- The front matter answered "yes" to the question of whether the data are available, but the instructions specified that "by request" should be treated as a "no" answer with an explanation. In response, we have revised the front matter to state "data available by request" and clarified this in the paper as well.

Both the front matter and the paper initially stated that there were no known competing financial interests, followed by detailed disclosures of potential competing interests. To eliminate any contradictions, we have removed the general "no conflict of interest" statement in the front matter and ensured that the detailed disclosures are the sole representation of any potential conflicts.

(2) The section "Cohort selection" is not sufficiently clear. I strongly recommend making a flowchart. Classes of patients are mentioned without saying whether they were included or excluded. For example, the text mentions 18 patients who were in other trials, and 74 patients who had a complete pathological response. I think the 18 were included and the 74 were excluded but this is not stated. It is also unclear throughout this section what the denominator of the given percentages is. For example, 11 patients had recurrence before adjuvant treatment: this is given as 5%, but 5% of what? Total patients? Eligible patients? Why are these 11 separated from "the main reasons for not starting adjuvant treatment"?--isn't recurrence a reason for not starting adjuvant treatment?

The trial protocol does a better job with this; I recommend basing the publication version on the protocol.

*Thank you for your valuable feedback. We appreciate your suggestion to clarify the Cohort Selection section. A proportion of patients received treatments other than nCRT alone and were therefore excluded, including the 18 patients who participated in clinical trials with additional systemic therapies. Regarding the 11 patients who had recurrence before starting treatment (5% of total of 229 patients assessed for eligibility), they are indeed part of the main reasons for not starting adjuvant treatment. The text in the results section was revised to make this more clear.*

*We acknowledge that the use of the term "eligible" for all patients who underwent surgery may cause confusion. We have revised the manuscript to align more closely with the terminology used in the Checkmate 577 trial, using "assessed for eligibility" instead. Additionally, we have updated Figure 1 (the flowchart) to better illustrate the cohort selection process and ensure clarity on the inclusion and exclusion criteria.*

(3) A number of abbreviations are used without explanation: someone deeply involved with this specific cancer will know them, but cancer researchers from other subdisciplines may not, and general readers definitely will not. In one case two different unexplained abbreviations turn out to refer to the same thing.

*We apologize for this oversight. We have carefully reviewed the manuscript and ensured that all abbreviations are defined at first use and used consistently throughout the text. Where appropriate, we have spelled out abbreviations to improve clarity for readers from other disciplines.*

Minor points:
- p. 8 "as advocated in the Checkmate 577" Strange choice of words: is "mandate" or "recommend" intended?

*Changed to “ recommend”*
- p. 295 "discolures" for "disclosures"

*Changed to “disclosures”*
- p. 10 clarify whether CPS was based on specimen analysis previously carried out by others (who? when? with what criteria?) or by the current researchers.

*Thank you for your comment. When available, the CPS score, as assessed during routine clinical care when indicated, was extracted from the data. No separate analysis was performed specifically for this study. This has been clarified in the methods section as follows (Method section, page 4):*

*“When available, the CPS (Combined Positive Score) determined from pre-treatment biopsies or surgical specimens was extracted from patient records. The score was assessed in accordance with local practice and clinical care guidelines as part of standard care when indicated.”*

- p. 10 Some patients apparently had recurrence before their first dose of nivolumab (and were therefore not dosed). What was the DFS value for these patients? Zero? Or were they excluded from DFS calculation?

*Thank you for your question. Patients who experienced recurrence before their first dose of nivolumab were not included in the DFS analysis, as disease-free survival was calculated from the start of adjuvant nivolumab treatment. The index date for DFS was the initiation of nivolumab, and only patients who received at least one dose were included in the DFS calculation.*

*However, we did describe recurrences occurring before the start of nivolumab in the manuscript, as these cases were relevant for evaluating the value of the baseline CT scan. This allowed us to assess whether baseline imaging could detect early recurrences prior to treatment initiation. We tried to make this more clear by adding the following sentences to the revised manuscript:*

- *Method section (page 5):* *“Secondary outcomes included disease-free survival (DFS) and diagnostic yield of routine CT scans per time point. DFS, defined as the time (in months) from the start of adjuvant nivolumab to disease recurrence or death, whichever occurred first. Adjuvant nivolumab was initiated within 16 weeks after esophagectomy for every patient, per protocol. Unless otherwise specified, all timepoints and analyses are referenced from the start of adjuvant nivolumab.”*
- *Table 3 (page 17): “Time (months from nivolumab initiation”*

- p. 11 "were summarized as means with standard deviations or medians with interquartile ranges" -- I am concerned this represents "experimenter degrees of freedom." Why were two different approaches used?

*Thank you for your comment. To clarify our approach, we have revised the manuscript to specify that means and standard deviations were used for normally distributed continuous variables, while medians and interquartile ranges were used for non-normally distributed variables. This method is consistent with standard statistical practice. Categorical variables were summarized as counts and percentages, and disease recurrence was reported descriptively. We added the following sentence (Method section, page 5):*

*“Continuous variables were summarized as mean with standard deviation (SD) when normally distributed, or medians with interquartile ranges (IQR) for non-normally distributed variables. Categorical variables were summarized as counts and percentages. Disease recurrence was reported descriptively.”*

- p. 11 "real-world cohort and the CheckMate 577 trial" I would be comfortable contrasting simulated data with real-world data, but this retrospective trial and the prospective CheckMate were both done in the real world with real patients. I recommend "retrospective" or simply "this study."

*Thank you for this suggestion, we changed it accordingly to “retrospective study” (page 9 and 10) and “this study” (page 5).*
